# Hospital capacities and shortages of healthcare resources among US hospitals during the coronavirus disease 2019 (COVID-19) pandemic, National Healthcare Safety Network (NHSN), March 27–July 14, 2020

**DOI:** 10.1017/ice.2021.280

**Published:** 2021-06-24

**Authors:** Hsiu Wu, Minn M. Soe, Rebecca Konnor, Raymund Dantes, Kathryn Haass, Margaret A. Dudeck, Cindy Gross, Denise Leaptrot, Mathew R. P. Sapiano, Katherine Allen-Bridson, Lauren Wattenmaker, Kelly Peterson, Kent Lemoine, Sheri Chernetsky Tejedor, Jonathan R. Edwards, Daniel Pollock, Andrea L. Benin

**Affiliations:** 1 Division of Healthcare Quality Promotion, Centers for Disease Control and Prevention, Atlanta, Georgia; 2 Division of Hospital Medicine, Department of Medicine, Emory University School of Medicine, Atlanta Georgia; 3 CACI, Atlanta, Georgia

## Abstract

During March 27–July 14, 2020, the Centers for Disease Control and Prevention’s National Healthcare Safety Network extended its surveillance to hospital capacities responding to COVID-19 pandemic. The data showed wide variations across hospitals in case burden, bed occupancies, ventilator usage, and healthcare personnel and supply status. These data were used to inform emergency responses.

The coronavirus disease 2019 (COVID-19) pandemic produced unprecedented stresses on hospitals in the United States in 2020.^1,2^ To create a national level of insight into the availability of hospital beds and resources, the Centers for Disease Control and Prevention’s National Healthcare Safety Network (NHSN), the nation’s healthcare surveillance system, launched a module for COVID-19 data collection on March 27, 2020.^2^ This module enabled voluntary reporting of patient counts, bed occupancies, and use of mechanical ventilators. Our previous report described the use of these data to generate imputed, survey-weighted estimates to express overall national healthcare capacity.^2^ On April 14, the NHSN added data elements for shortages of healthcare personnel (HCP) and healthcare supplies including personal protective equipment (PPE). In this report, to establish a baseline from which future data collection can be built, we describe previously unreported (1) raw, hospital-level occupancy of beds and of ventilators, (2) national trends by facility type, (3) shortages of HCP and PPE, and (4) use of the data by the public health response.

## Methods

We obtained the data colledted by the NHSN from March 27 through July 14, 2020. We chose limited metrics of hospital capacity based on decisions about the likely most important information obtainable with manageble burden. These metrics were intended to measure the national landscape in a standardized fashion and were not expected to measure the full complement of what an individual hospital or locality would need to manage the pandemic.

We calculated the distribution of hospital-level capacities by month. For each month, we used data for the day when the highest number of patients with clinically suspected or laboratory-confirmed COVID-19 was reported for each hospital. If the highest number occurred on multiple days, the last day was used.

The temporal changes of hospital capacities were analyzed from April 13 through July 13. Using generalized log-linear mixed models, we regressed the effect of time on daily percent change of individual measures while adjusting for differential distributions of number of beds and hospital type and daily participation rate (%) over time.^3^

We describe the reported shortages of HCP, PPE, or ventilator supplies for ≥1 day during April 14–July 14, 2020. HCP shortages were defined as a critical staffing shortage reported by the hospital (https://www.cdc.gov/nhsn/pdfs/covid19/archive/57.131-toi-apr13-508.pdf). Supply shortages were defined reporting no on-hand supply for one of the categories of PPE or ventilator supplies including flow sensors, tubing, connectors, valves, and filters (https://www.cdc.gov/nhsn/pdfs/covid19/archive/57.132-toi-jul2-508.pdf).

## Results

### Distribution of hospital capacities

Among 6,194 hospitals enrolled in NHSN during March 27–July 14, 4,535 (73%) reported data on hospitalized COVID-19 patients (Supplementary Table 1 online). Hospitals had from 0 to 694 inpatients with COVID-19. The median percentage of inpatient beds occupied by patients with COVID-19 was highest (7.8%) in April, when one-quarter of reporting hospitals had >15% of beds occupied by patients with COVID-19. During this time, 6%–9% hospitals had >60% of their ventilators in use. The highest use of ventilators (median, 12.3%) occurred in April when a quarter of hospitals had >76% in-use ventilators used for patients with COVID-19 (Table 1).

**Table 1.** Distribution of Hospital-Level Patient Counts, Hospital Bed Occupancies, and Ventilator Use on the Day When the Highest Number of COVID-19 Patients Was Reported Per Hospital in 4 Time Periods—National Healthcare Safety Network, March 27–July 14, 2020

Variable	March 27–April 30	May	June	July 1–July 14
No. of reporting hospitals	4,167	4,020	4,030	3,776
Hospitalized patients with COVID-19^a^ per hospital, range, median (IQR)	0–694, 5 (1–20)	0–421, 5 (1–19)	0–260, 5 (1–19)	0–313, 4 (1–19)
COVID-19^a^ inpatients on mechanical ventilators, no., median % (IQR)^b^	3,074, 4.1 (0–18.2)	2,984, 0 (0–14.8)	2,757, 0 (0–13.8)	2,436, 0 (0–13.3)
Inpatient beds occupied, no., median % (IQR)	3,906, 50 (31.3–67.4)	3,838, 54.2 (34.4–71.1)	3,846, 59.2 (38.3–76.7)	3,595, 60.0 (38.9–78.1)
Hospitals with ≥80% of inpatient beds occupied, no., (%)	555 (13.4)	612 (15.3)	828 (20.6)	820 (21.8)
Inpatient beds occupied by COVID-19 patients, no., median % (IQR)	4,152, 7.8 (3.3–15.6)	4,007, 7.7 (2.9–15.4)	4,021, 6.8 (2.5–13.1)	3,763, 5.9 (1.6–13.6)
ICU beds occupied, no., median % (IQR)^c^	2,628, 62.5 (40.0–80.6)	2,570, 61.9 (40.0–81.8)	2,453, 63.6 (41.7–83.3)	2,281, 65.0 (42.5–83.3)
Hospitals with ≥80% ICU beds occupied, no. (%)^c^	707 (26.6)	713 (27.6)	734 (29.8)	701 (30.5)
Ventilators in use, no., median % (IQR)^d^	3,253, 12.3 (0–33.3)	3,225, 9.5 (0–29.0)	3,284, 10.5 (0–28.1)	3,081, 10.9 (0–29.6)
Hospitals with ≥60% of ventilators in use, no. (%)^d^	277 (8.5)	231 (7.1)	211 (6.4)	222 (6.2)
In-use ventilators occupied by COVID-19 patients, no., median % (IQR)^e^	1,979, 38.1 (0–76.9)	1,930, 29.2 (0–66.7)	1,810, 23.7 (0–50)	1,687, 19.4 (0–50)

Note. IQR, interquartile range; COVID-19, coronavirus disease 2019; ICU, intensive care unit. For the measures with a response rate <100%, the no. of hospitals that contributed to the measure is provided.

a Suspected or confirmed.

b Only includes hospitals that reported ≥1 hospitalized COVID-19 patients and ≥1 ventilator. No. of hospitals that met this criteria: 3,192, 3,049, 3,012, and 2,693 in each period.

c Only includes hospitals that reported ≥1 ICU bed. No. of hospitals that met this criteria: 2,660, 2,581, 2,464, and 2,295 in each period.

d Only includes hospitals that reported ≥1 ventilator. No. of hospitals that met this criteria: 3,275, 3,251, 3,310, and 3,107 in each period.

e Only includes hospitals that reported ≥1 ventilator in use. No. of hospitals that met this criteria: 2,027, 1,968, 2,024, and 1,906 in each period.

### Temporal changes of hospital capacities

**Fig. 1.**
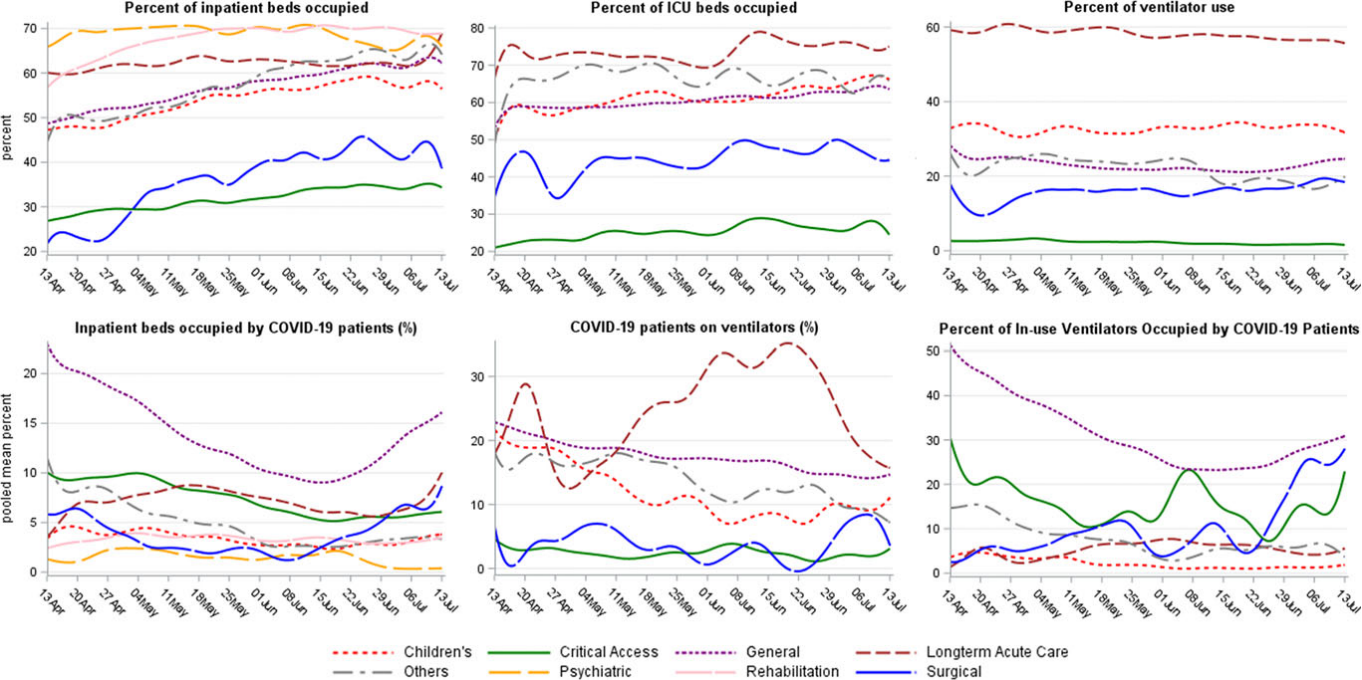
COVID-19 impact measures on hospital capacity, trends by hospital type —National Healthcare Safety Network, April 13–July 13, 2020. *Number of reporting hospitals vary by hospital type (see Supplementary Table 1 online).

Figure 1 shows the temporal changes in metrics of hospital capacity. The adjusted percentage of inpatient beds occupied by patients with COVID-19 decreased from 21% to 8% by June 15 and then increased again after June 16. Among hospitals with mechanical ventilators, overall adjusted ventilator use was ∼25%; it was highest among long-term acute care hospitals (∼60%) and lowest among critical access hospitals (∼5%).

### Shortages of healthcare resources

From April 14 to July 14, among 2,349 hospitals reporting staffing status, 676 hospitals (29%) reported immediate shortages of HCP, including shortages of nurses (n = 372 hospitals), respiratory therapists (n = 250), environmental services staff (n = 217), physicians (n = 138), pharmacists (n = 125), and temporary workers (n = 157). A range of COVID-19 bed occupancies (0%–12%; median, 3%) were reported concurrently.

Of 3,145 hospitals reporting status of supplies, 362 (11%) reported having no on-hand supply for at least 1 day, including eye protection (face shields or goggles; n = 125 hospitals), single-use gowns (n = 103), ventilator supplies (n = 101), N95 respirators (n = 80), surgical masks (n = 64), and gloves (n = 64). A range of bed occupancies of patients with COVID-19 (0%–90%; median, 3%) were reported concurrently.

### Use of NHSN in public health response

The NHSN data-sharing functionality enabled hospitals to share data with state and local health departments in real time.^4^ Also, because of the HAI reporting requirements for hospitals that were introduced in 2010 by the Centers for Medicare & Medicaid Services, hospital users were already familiar with NHSN interface and analytic tools. These features contributed to the rapid uptake of the NHSN COVID-19 surveillance platform among hospitals. During the period of the data collection, the NHSN team provided daily summaries and visualizations of the data to CDC emergency operations, the Department of Health and Human Services (including the Office of the Assistant Secretary for Preparedness and Response), the Federal Emergency Management Agency, and state or territory health departments. These data were used federally for situational awareness and resource allocation. For example, data on PPE were used by the federal supply chain visualization and planning program. A public-facing data dashboard and data set were available online.^5^

## Discussion

From late March to early July 2020, the NHSN collected data on the national trends of capacity of inpatient beds and ventilators as well as shortages of supplies and personnel for the purposes of providing standardized, national data to support healthcare in the pandemic. The early phase of the pandemic highlighted the pre-existing lack of national visibility into the healthcare system; and this data collection demonstrated the powerful potential of integrating such data collection into healthcare surveillance using the NHSN platform.

The results of evaluating the national trends of hospital bed occupancies and COVID-19 patients on ventilators using regression methods agreed with the time-series estimates of capacity we reported previously.^2^ However, all capacity indicators showed significant variations across hospitals. Almost one-third of reporting hospitals had an immediate shortage of HCP, and 11% faced shortages of PPE or ventilator supplies. Shortages of HCP occurred in hospitals regardless of volume of patients with COVID-19. Possible causes include baseline shortages, mounting incidence of COVID-19 among HCPs, HCP requiring quarantine due to exposure to SARS CoV-2,^6^ or HCP taking on care responsibilities for family members. Shortages of nurses, an important factor in patient mortality,^7^ were reported in ˜16% hospitals. Also, regardless the numbers of COVID-19 patients, many hospitals experienced shortages of PPE or supplies for ventilators, reflecting both inadequate reserves and interrupted supply chains.^8,9^

This study had several limitations. First, there was no federal COVID-19 reporting mandate during the study period. Hospital reporting fluctuated daily, potentially affecting the precision of calculations. We mitigated this limitation by regression adjustment with random effects. Second, we did not distinguish between numbers of patients with clinically suspected and laboratory-confirmed COVID-19. Third, we did not collect information on patients with COVID-19 requiring critical care or other risk factors for mortality.^10^ Fourth, no baseline information was available on national hospital capacity, supplies, or HCP to use for comparison. The need for such comparison became readily apparent and underscores the importance of collecting such data during noncrisis periods.

The public health requirements for information during the pandemic highlighted the need for a surveillance system that can generate baseline data as well as swiftly gather data and share it with various emergency response entities during a crisis. The NHSN was able to fill this gap in the early phase of the COVID-19 pandemic.

## Supplementary material

For supplementary material accompanying this paper visit http://dx.doi.org/10.1017/ice.2021.280. click here to view supplementary material
