# Supplementary material for: Hospital capacities and shortages of healthcare resources among US hospitals during the coronavirus disease 2019 (COVID-19) pandemic, National Healthcare Safety Network (NHSN), March 27–July 14, 2020
Source: Infect Control Hosp Epidemiol. 2021 Jun 24:1–4. doi: 10.1017/ice.2021.280 (PMC8280389; doi:10.1017/ice.2021.280)
Supplement: Supplementary file 1 [file S0899823X21002804sup001.docx]

**Supplemental Table.** Numbers and characteristics of reporting hospitals—National Healthcare Safety Network

|  | Hospitals actively enrolled in NHSN  (n=6194) | Hospitals reporting to COVID-19 module  (n=4535) | Hospitals in trend analysis  (n=4198) |
| --- | --- | --- | --- |
| Hospital type |  |  |  |
| General | 3516 | 2725 | 2612 |
| Critical Access | 1227 | 977 | 914 |
| Long-term Acute care | 449 | 214 | 204 |
| Rehabilitation | 371 | 245 | 240 |
| Psychiatric | 152 | 35 | 31 |
| Veterans Affairs | 127 | 126 | N/A |
| Surgical | 127 | 81 | 72 |
| Children's | 107 | 71 | 70 |
| Others* | 118 | 61 | 55 |
|  |  |  |  |
| Bed size |  |  |  |
| >500 | 242 | 185 | 175 |
| 301–500 | 545 | 447 | 422 |
| 201–300 | 604 | 474 | 450 |
| 101–200 | 1053 | 819 | 763 |
| 51–100 | 1038 | 754 | 697 |
| 26–50 | 1024 | 661 | 607 |
| ≤25 | 1653 | 1195 | 1073 |
| Missing | 35 | 42 | 11 |

N/A, not applicable

*Include oncology, orthopedic, women’s, women and children’s, military hospitals
